# Special Issue: Carbon-Based Nanomaterials for (Bio)Sensors Development

**DOI:** 10.3390/nano11092430

**Published:** 2021-09-18

**Authors:** Simone Morais

**Affiliations:** REQUIMTE–LAQV, Instituto Superior de Engenharia, Instituto Politécnico do Porto, R. Dr. António Bernardino de Almeida 431, 4249-015 Porto, Portugal; sbm@isep.ipp.pt

Carbon-based nanomaterials have been increasingly used in the design of sensors and biosensors due to their advantageous intrinsic properties, which include, but are not limited to, high electrical and thermal conductivity, chemical stability, optical properties, large specific surface, biocompatibility, and easy functionalization. Therefore, the final aim of this Special Issue is to share new data concerning the novel exploitation strategies of these nanomaterials in order to support the development of improved (bio)sensing tools. Focus is mostly placed on the usage of graphene but also on carbon dots and carbon nanotubes, as well as on the preparation and characterization of new (nano)composites. The tailoring of the (bio)sensor surface is the common approach of the different reported schemes when optimizing the (bio)sensor design. Simulation tests are also performed [1].

The research community has shown rising and commendable interest in searching for and applying greener synthesis methodologies, with different studies [2,3,4] exploring novel pathways, e.g., the preparation of carbon dots from microalgae and water [2]. Sustainable routes for nanomaterials’ synthesis clearly constitute research opportunities while contributing to their low-cost production, wide use, and, obviously, circular economy.

Moreover, for those researchers seeking an overview of the state of the art of the use of carbon-based nanomaterials for (bio)sensors’ development, three review papers targeting different topics are included in this Special Issue. Pan et al. [5] revised, in detail, the design of chemical sensors and biosensors for a food safety assessment. Emphasis was placed on the role of (single- and multi-walled) carbon nanotubes, graphene, and carbon quantum dots in increasing (bio)sensor sensitivity, accuracy and precision, and detection capacity for pesticides’ residues, veterinary pharmaceutical compounds, adulterants, methylmercury, mycotoxins, and hormones, among others, in foodstuff. Moreover, the tremendous potential of carbonaceous nanomaterials (graphene, carbon nanotubes, carbon nanopowder, fullerene, carbon nanofibers, etc.) in the modification of electrochemical (bio)sensor surfaces toward the detection of contaminants of emerging concern (specifically, pharmaceutical pollutants, such as antibiotics, anticonvulsants, antidiabetics, anti-inflammatory drugs, hormones, β-blockers, etc.) in waters and marine species was also critically discussed by Torrinha et al. [6]. These authors highlighted the undeniable contribution of carbon nanomaterials to the miniaturization and portability of the (bio)sensors besides the huge impact on their electroanalytical performance. In a broader context, in terms of the fields of application (as macro- and small molecules, gas, strain/pressure sensors), Wang et al. [7] comprehensively reviewed the synthesis techniques for carbon nanofiber-based nanomaterials, including their functionalization with polymers, metal oxide nanoparticles, silica, etc. The prospects for novel applications in fields such as energy, catalysis, and environmental science were also identified.

The incorporation of carbon-based nanomaterials, independent of the detection scheme and developed platform type (mechanical, thermal, optical, magnetic, chemical, and biological), has demonstrated a major beneficial effect on the sensitivity, specificity, and overall performance of (bio)sensors. Consequently, carbon-based nanomaterials have brought about a revolution in the field of (bio)sensors with the development of increasingly sensitive devices.
